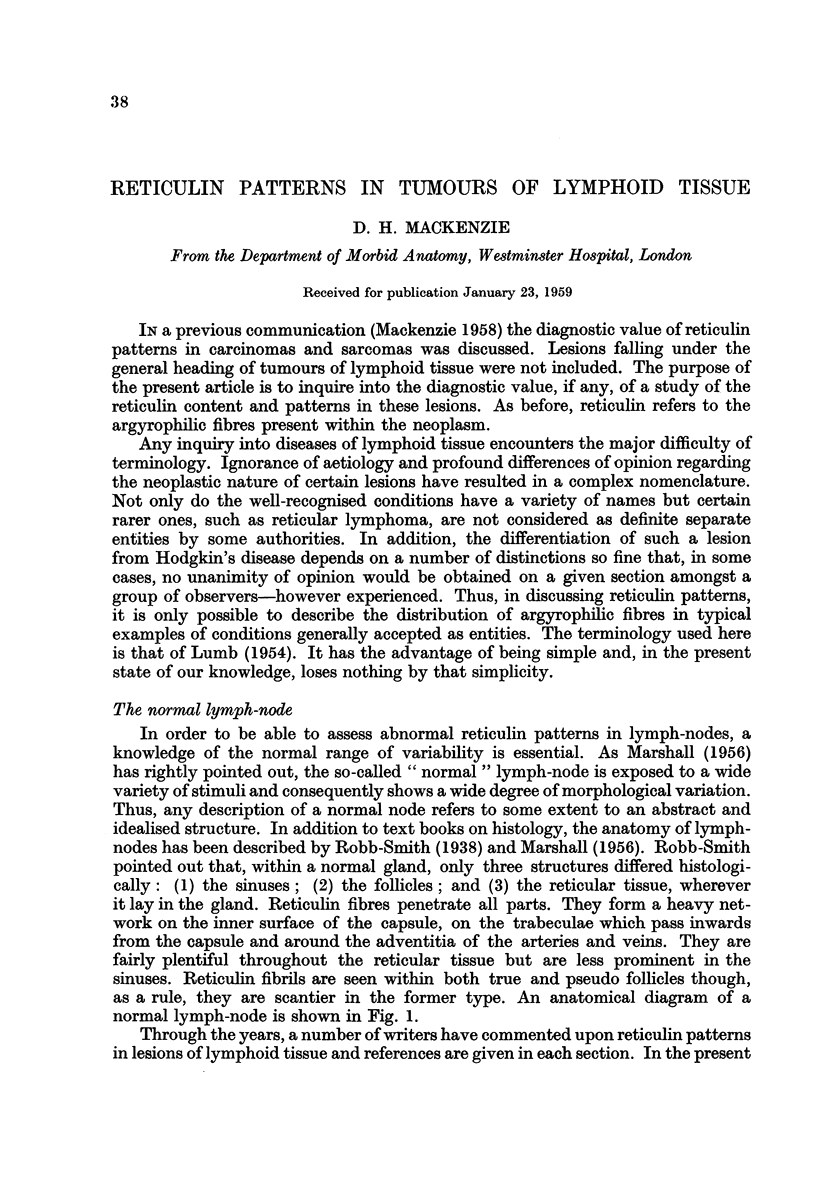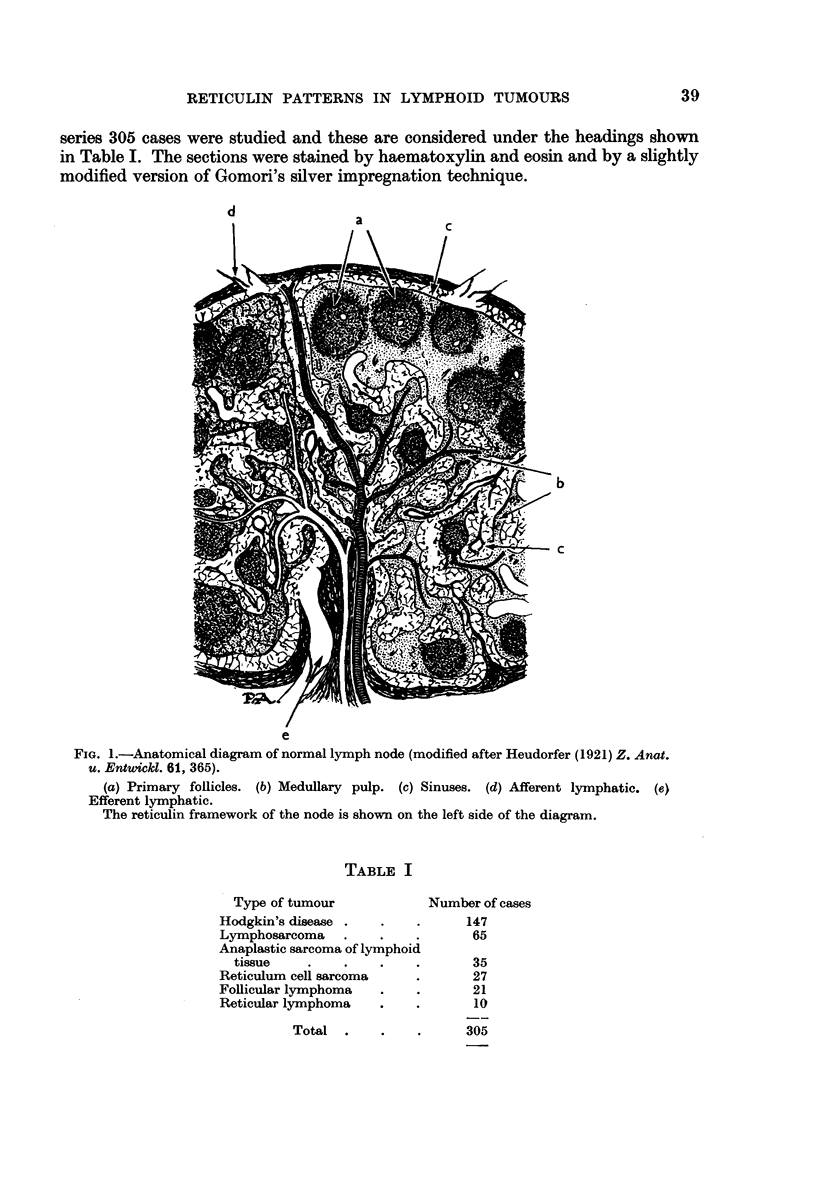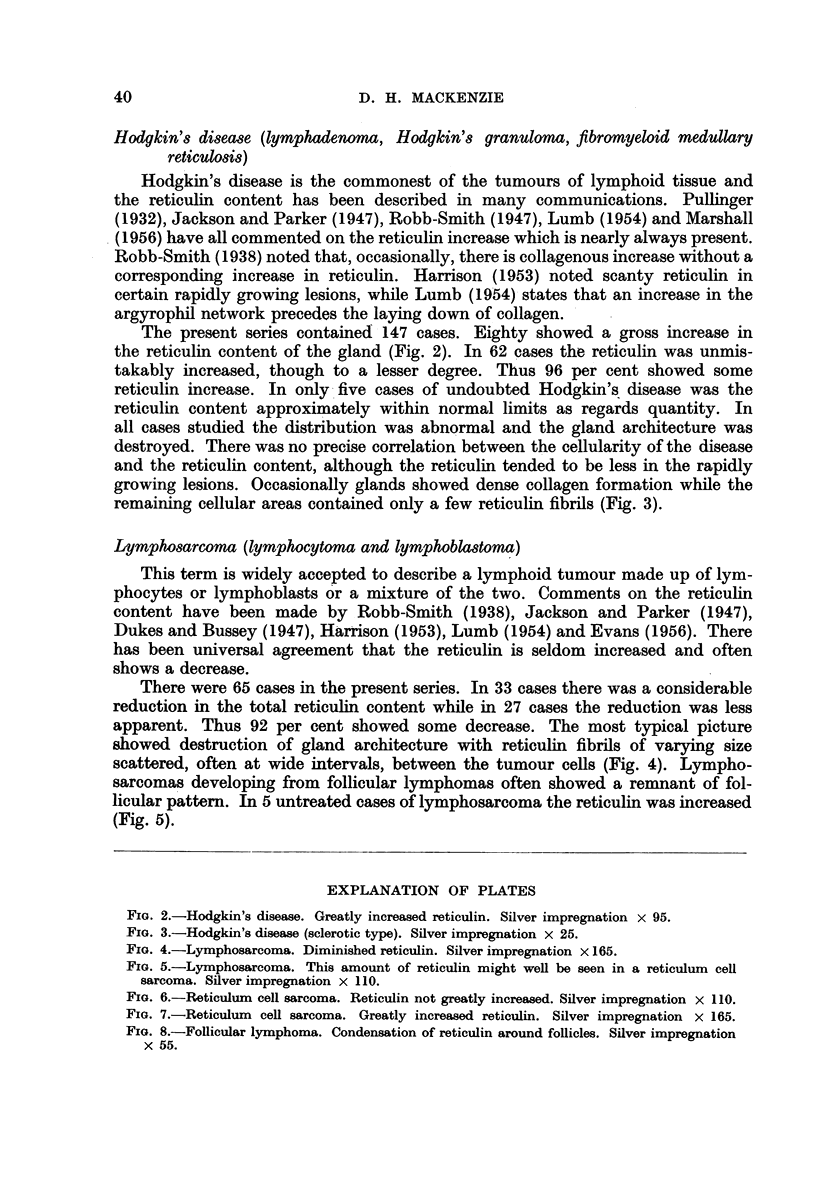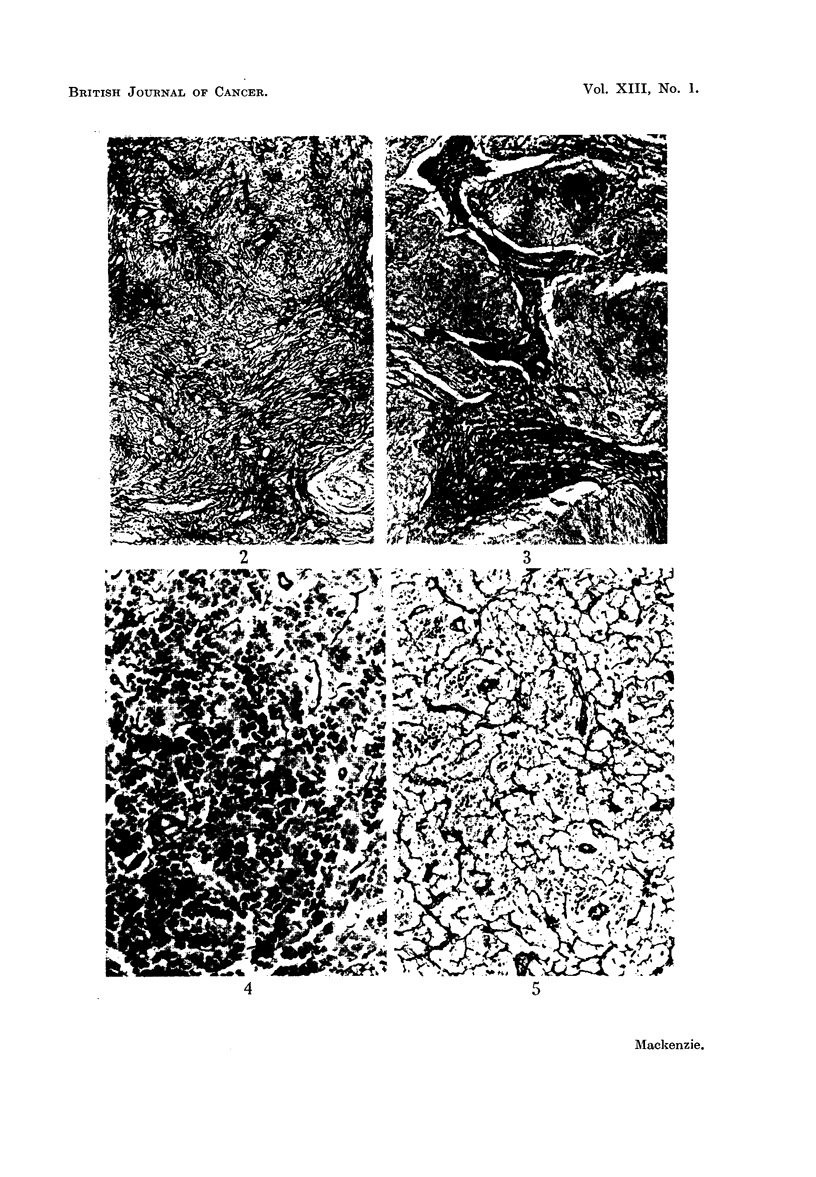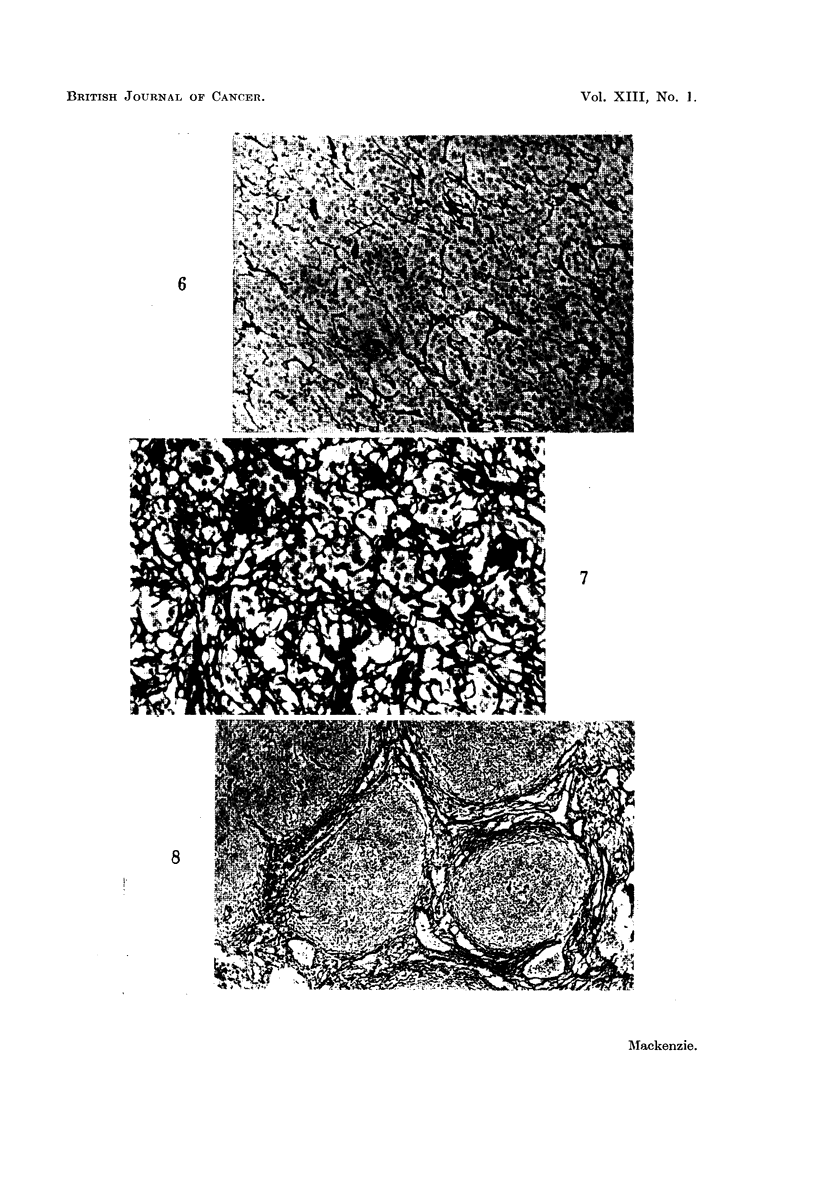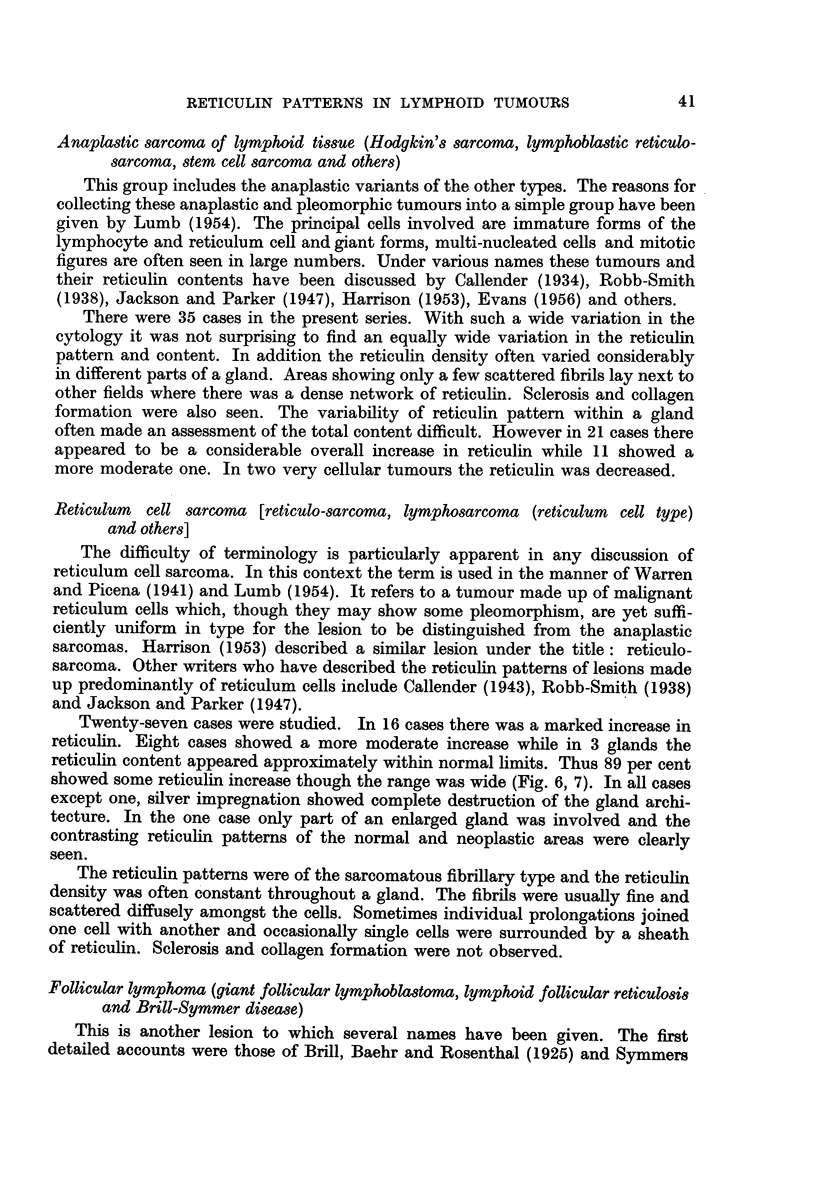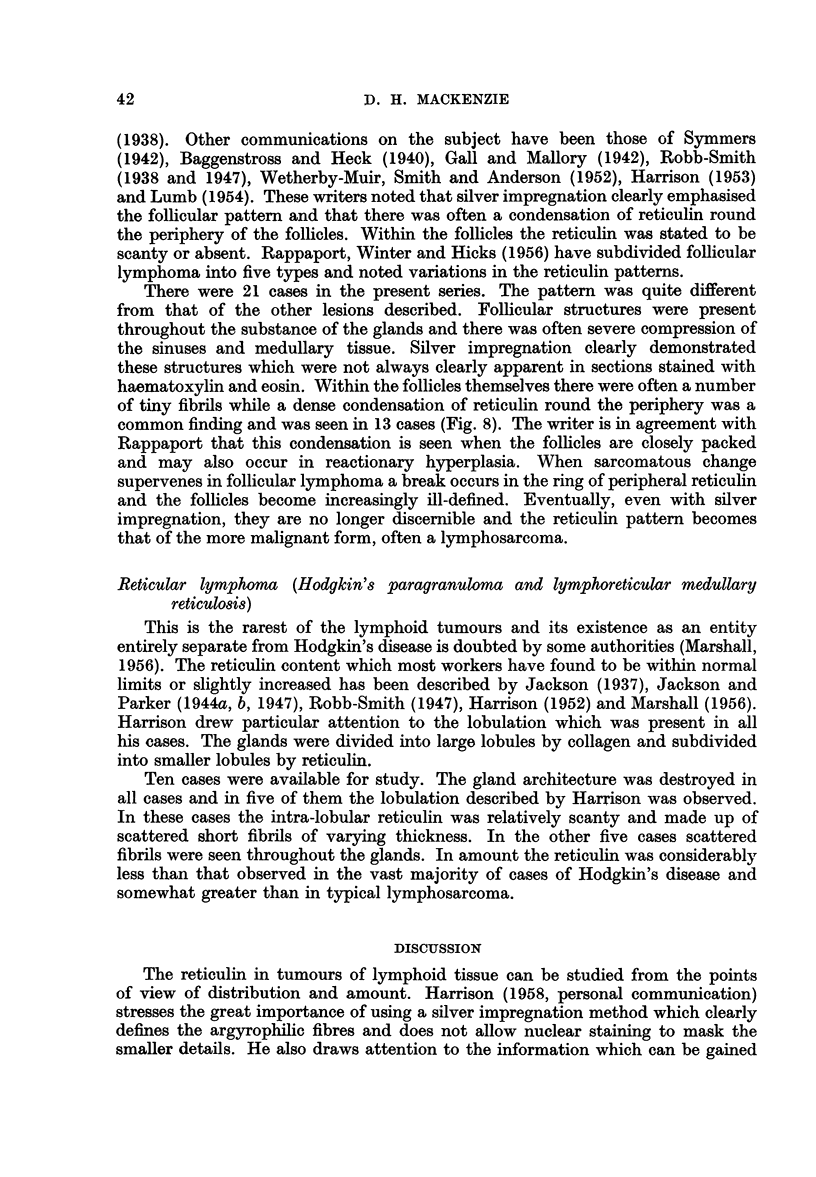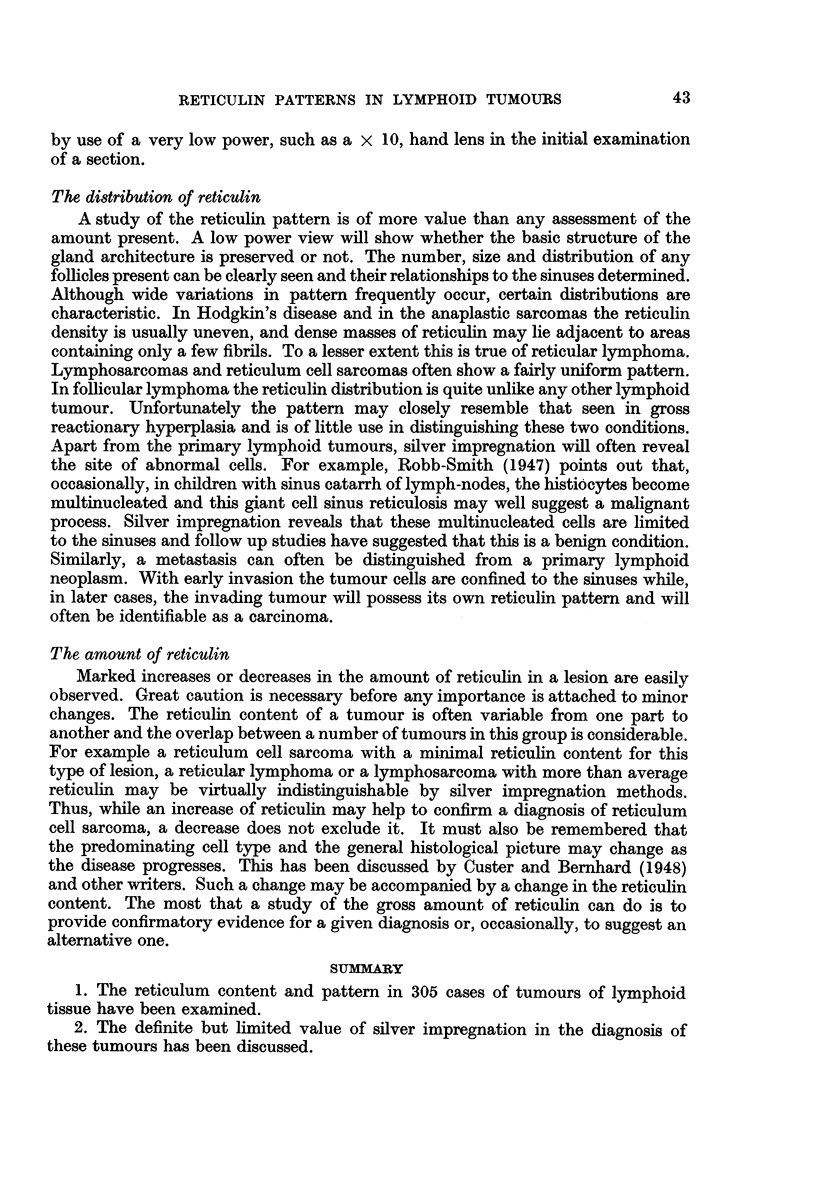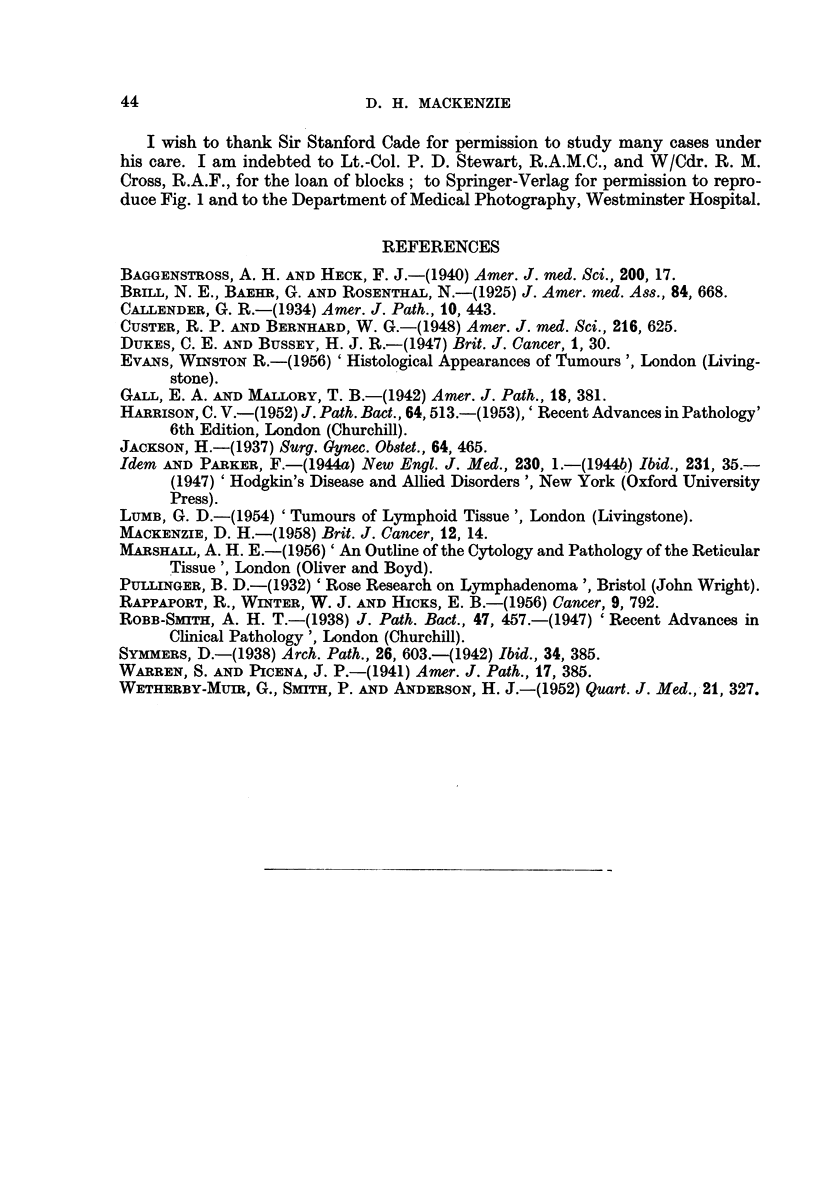# Reticulin Patterns in Tumours of Lymphoid Tissue

**DOI:** 10.1038/bjc.1959.6

**Published:** 1959-03

**Authors:** D. H. Mackenzie

## Abstract

**Images:**


### RETICULIN PATTERNS IN TUMOURS OF LYMPHOID TISSUE

D. H. MACKENZIE

*From the Department of Morbid Anatomy, Westminster Hospital, London*

Received for publication January 23, 1959

IN a previous communication (Mackenzie 1958) the diagnostic value of reticulin patterns in carcinomas and sarcomas was discussed. Lesions falling under the general heading of tumours of lymphoid tissue were not included. The purpose of the present article is to inquire into the diagnostic value, if any, of a study of the reticulin content and patterns in these lesions. As before, reticulin refers to the argyrophilic fibres present within the neoplasm.

Any inquiry into diseases of lymphoid tissue encounters the major difficulty of terminology. Ignorance of aetiology and profound differences of opinion regarding the neoplastic nature of certain lesions have resulted in a complex nomenclature. Not only do the well-recognised conditions have a variety of names but certain rarer ones, such as reticular lymphoma, are not considered as definite separate entities by some authorities. In addition, the differentiation of such a lesion from Hodgkin's disease depends on a number of distinctions so fine that, in some cases, no unanimity of opinion would be obtained on a given section amongst a group of observers—however experienced. Thus, in discussing reticulin patterns, it is only possible to describe the distribution of argyrophilic fibres in typical examples of conditions generally accepted as entities. The terminology used here is that of Lumb (1954). It has the advantage of being simple and, in the present state of our knowledge, loses nothing by that simplicity.

### *The normal lymph-node*

In order to be able to assess abnormal reticulin patterns in lymph-nodes, a knowledge of the normal range of variability is essential. As Marshall (1956) has rightly pointed out, the so-called " normal " lymph-node is exposed to a wide variety of stimuli and consequently shows a wide degree of morphological variation. Thus, any description of a normal node refers to some extent to an abstract and idealised structure. In addition to text books on histology, the anatomy of lymph-nodes has been described by Robb-Smith (1938) and Marshall (1956). Robb-Smith pointed out that, within a normal gland, only three structures differed histologically : (1) the sinuses ; (2) the follicles ; and (3) the reticular tissue, wherever it lay in the gland. Reticulin fibres penetrate all parts. They form a heavy network on the inner surface of the capsule, on the trabeculae which pass inwards from the capsule and around the adventitia of the arteries and veins. They are fairly plentiful throughout the reticular tissue but are less prominent in the sinuses. Reticulin fibrils are seen within both true and pseudo follicles though, as a rule, they are scantier in the former type. An anatomical diagram of a normal lymph-node is shown in Fig. 1.

Through the years, a number of writers have commented upon reticulin patterns in lesions of lymphoid tissue and references are given in each section. In the present series 305 cases were studied and these are considered under the headings shown in Table I. The sections were stained by haematoxylin and eosin and by a slightly modified version of Gomori's silver impregnation technique.

FIG. 1.—Anatomical diagram of normal lymph node (modified after Heudorfer (1921) *Z. Anat. u. Entwickl.* **61**, 365).

(*a*) Primary follicles. (*b*) Medullary pulp. (*c*) Sinuses. (*d*) Afferent lymphatic. (*e*) Efferent lymphatic.

The reticulin framework of the node is shown on the left side of the diagram.

TABLE I

| Type of tumour | Number of cases |
|---|---|
| Hodgkin's disease . . . | 147 |
| Lymphosarcoma . . . | 65 |
| Anaplastic sarcoma of lymphoid tissue . . . . | 35 |
| Reticulum cell sarcoma . | 27 |
| Follicular lymphoma . . | 21 |
| Reticular lymphoma . . | 10 |
| Total . . . | 305 |

*Hodgkin's disease (lymphadenoma, Hodgkin's granuloma, fibromyeloid medullary reticulosis)*

Hodgkin's disease is the commonest of the tumours of lymphoid tissue and the reticulin content has been described in many communications. Pullinger (1932), Jackson and Parker (1947), Robb-Smith (1947), Lumb (1954) and Marshall (1956) have all commented on the reticulin increase which is nearly always present. Robb-Smith (1938) noted that, occasionally, there is collagenous increase without a corresponding increase in reticulin. Harrison (1953) noted scanty reticulin in certain rapidly growing lesions, while Lumb (1954) states that an increase in the argyrophil network precedes the laying down of collagen.

The present series contained 147 cases. Eighty showed a gross increase in the reticulin content of the gland (Fig. 2). In 62 cases the reticulin was unmistakably increased, though to a lesser degree. Thus 96 per cent showed some reticulin increase. In only five cases of undoubted Hodgkin's disease was the reticulin content approximately within normal limits as regards quantity. In all cases studied the distribution was abnormal and the gland architecture was destroyed. There was no precise correlation between the cellularity of the disease and the reticulin content, although the reticulin tended to be less in the rapidly growing lesions. Occasionally glands showed dense collagen formation while the remaining cellular areas contained only a few reticulin fibrils (Fig. 3).

*Lymphosarcoma (lymphocytoma and lymphoblastoma)*

This term is widely accepted to describe a lymphoid tumour made up of lymphocytes or lymphoblasts or a mixture of the two. Comments on the reticulin content have been made by Robb-Smith (1938), Jackson and Parker (1947), Dukes and Bussey (1947), Harrison (1953), Lumb (1954) and Evans (1956). There has been universal agreement that the reticulin is seldom increased and often shows a decrease.

There were 65 cases in the present series. In 33 cases there was a considerable reduction in the total reticulin content while in 27 cases the reduction was less apparent. Thus 92 per cent showed some decrease. The most typical picture showed destruction of gland architecture with reticulin fibrils of varying size scattered, often at wide intervals, between the tumour cells (Fig. 4). Lymphosarcomas developing from follicular lymphomas often showed a remnant of follicular pattern. In 5 untreated cases of lymphosarcoma the reticulin was increased (Fig. 5).

---

EXPLANATION OF PLATES

FIG. 2.—Hodgkin's disease. Greatly increased reticulin. Silver impregnation × 95.

FIG. 3.—Hodgkin's disease (sclerotic type). Silver impregnation × 25.

FIG. 4.—Lymphosarcoma. Diminished reticulin. Silver impregnation ×165.

FIG. 5.—Lymphosarcoma. This amount of reticulin might well be seen in a reticulum cell sarcoma. Silver impregnation × 110.

FIG. 6.—Reticulum cell sarcoma. Reticulin not greatly increased. Silver impregnation × 110.

FIG. 7.—Reticulum cell sarcoma. Greatly increased reticulin. Silver impregnation × 165.

FIG. 8.—Follicular lymphoma. Condensation of reticulin around follicles. Silver impregnation × 55.

2

3

4

5

Mackenzie.

6

7

8

*Anaplastic sarcoma of lymphoid tissue (Hodgkin's sarcoma, lymphoblastic reticulo-sarcoma, stem cell sarcoma and others)*

This group includes the anaplastic variants of the other types. The reasons for collecting these anaplastic and pleomorphic tumours into a simple group have been given by Lumb (1954). The principal cells involved are immature forms of the lymphocyte and reticulum cell and giant forms, multi-nucleated cells and mitotic figures are often seen in large numbers. Under various names these tumours and their reticulin contents have been discussed by Callender (1934), Robb-Smith (1938), Jackson and Parker (1947), Harrison (1953), Evans (1956) and others.

There were 35 cases in the present series. With such a wide variation in the cytology it was not surprising to find an equally wide variation in the reticulin pattern and content. In addition the reticulin density often varied considerably in different parts of a gland. Areas showing only a few scattered fibrils lay next to other fields where there was a dense network of reticulin. Sclerosis and collagen formation were also seen. The variability of reticulin pattern within a gland often made an assessment of the total content difficult. However in 21 cases there appeared to be a considerable overall increase in reticulin while 11 showed a more moderate one. In two very cellular tumours the reticulin was decreased.

*Reticulum cell sarcoma [reticulo-sarcoma, lymphosarcoma (reticulum cell type) and others]*

The difficulty of terminology is particularly apparent in any discussion of reticulum cell sarcoma. In this context the term is used in the manner of Warren and Picena (1941) and Lumb (1954). It refers to a tumour made up of malignant reticulum cells which, though they may show some pleomorphism, are yet sufficiently uniform in type for the lesion to be distinguished from the anaplastic sarcomas. Harrison (1953) described a similar lesion under the title : reticulo-sarcoma. Other writers who have described the reticulin patterns of lesions made up predominantly of reticulum cells include Callender (1943), Robb-Smith (1938) and Jackson and Parker (1947).

Twenty-seven cases were studied. In 16 cases there was a marked increase in reticulin. Eight cases showed a more moderate increase while in 3 glands the reticulin content appeared approximately within normal limits. Thus 89 per cent showed some reticulin increase though the range was wide (Fig. 6, 7). In all cases except one, silver impregnation showed complete destruction of the gland architecture. In the one case only part of an enlarged gland was involved and the contrasting reticulin patterns of the normal and neoplastic areas were clearly seen.

The reticulin patterns were of the sarcomatous fibrillary type and the reticulin density was often constant throughout a gland. The fibrils were usually fine and scattered diffusely amongst the cells. Sometimes individual prolongations joined one cell with another and occasionally single cells were surrounded by a sheath of reticulin. Sclerosis and collagen formation were not observed.

*Follicular lymphoma (giant follicular lymphoblastoma, lymphoid follicular reticulosis and Brill-Symmer disease)*

This is another lesion to which several names have been given. The first detailed accounts were those of Brill, Baehr and Rosenthal (1925) and Symmers

(1938). Other communications on the subject have been those of Symmers (1942), Baggenstross and Heck (1940), Gall and Mallory (1942), Robb-Smith (1938 and 1947), Wetherby-Muir, Smith and Anderson (1952), Harrison (1953) and Lumb (1954). These writers noted that silver impregnation clearly emphasised the follicular pattern and that there was often a condensation of reticulin round the periphery of the follicles. Within the follicles the reticulin was stated to be scanty or absent. Rappaport, Winter and Hicks (1956) have subdivided follicular lymphoma into five types and noted variations in the reticulin patterns.

There were 21 cases in the present series. The pattern was quite different from that of the other lesions described. Follicular structures were present throughout the substance of the glands and there was often severe compression of the sinuses and medullary tissue. Silver impregnation clearly demonstrated these structures which were not always clearly apparent in sections stained with haematoxylin and eosin. Within the follicles themselves there were often a number of tiny fibrils while a dense condensation of reticulin round the periphery was a common finding and was seen in 13 cases (Fig. 8). The writer is in agreement with Rappaport that this condensation is seen when the follicles are closely packed and may also occur in reactionary hyperplasia. When sarcomatous change supervenes in follicular lymphoma a break occurs in the ring of peripheral reticulin and the follicles become increasingly ill-defined. Eventually, even with silver impregnation, they are no longer discernible and the reticulin pattern becomes that of the more malignant form, often a lymphosarcoma.

*Reticular lymphoma (Hodgkin's paragranuloma and lymphoreticular medullary reticulosis)*

This is the rarest of the lymphoid tumours and its existence as an entity entirely separate from Hodgkin's disease is doubted by some authorities (Marshall, 1956). The reticulin content which most workers have found to be within normal limits or slightly increased has been described by Jackson (1937), Jackson and Parker (1944*a*, *b*, 1947), Robb-Smith (1947), Harrison (1952) and Marshall (1956). Harrison drew particular attention to the lobulation which was present in all his cases. The glands were divided into large lobules by collagen and subdivided into smaller lobules by reticulin.

Ten cases were available for study. The gland architecture was destroyed in all cases and in five of them the lobulation described by Harrison was observed. In these cases the intra-lobular reticulin was relatively scanty and made up of scattered short fibrils of varying thickness. In the other five cases scattered fibrils were seen throughout the glands. In amount the reticulin was considerably less than that observed in the vast majority of cases of Hodgkin's disease and somewhat greater than in typical lymphosarcoma.

### DISCUSSION

The reticulin in tumours of lymphoid tissue can be studied from the points of view of distribution and amount. Harrison (1958, personal communication) stresses the great importance of using a silver impregnation method which clearly defines the argyrophilic fibres and does not allow nuclear staining to mask the smaller details. He also draws attention to the information which can be gained by use of a very low power, such as a × 10, hand lens in the initial examination of a section.

*The distribution of reticulin*

A study of the reticulin pattern is of more value than any assessment of the amount present. A low power view will show whether the basic structure of the gland architecture is preserved or not. The number, size and distribution of any follicles present can be clearly seen and their relationships to the sinuses determined. Although wide variations in pattern frequently occur, certain distributions are characteristic. In Hodgkin's disease and in the anaplastic sarcomas the reticulin density is usually uneven, and dense masses of reticulin may lie adjacent to areas containing only a few fibrils. To a lesser extent this is true of reticular lymphoma. Lymphosarcomas and reticulum cell sarcomas often show a fairly uniform pattern. In follicular lymphoma the reticulin distribution is quite unlike any other lymphoid tumour. Unfortunately the pattern may closely resemble that seen in gross reactionary hyperplasia and is of little use in distinguishing these two conditions. Apart from the primary lymphoid tumours, silver impregnation will often reveal the site of abnormal cells. For example, Robb-Smith (1947) points out that, occasionally, in children with sinus catarrh of lymph-nodes, the histiocytes become multinucleated and this giant cell sinus reticulosis may well suggest a malignant process. Silver impregnation reveals that these multinucleated cells are limited to the sinuses and follow up studies have suggested that this is a benign condition. Similarly, a metastasis can often be distinguished from a primary lymphoid neoplasm. With early invasion the tumour cells are confined to the sinuses while, in later cases, the invading tumour will possess its own reticulin pattern and will often be identifiable as a carcinoma.

*The amount of reticulin*

Marked increases or decreases in the amount of reticulin in a lesion are easily observed. Great caution is necessary before any importance is attached to minor changes. The reticulin content of a tumour is often variable from one part to another and the overlap between a number of tumours in this group is considerable. For example a reticulum cell sarcoma with a minimal reticulin content for this type of lesion, a reticular lymphoma or a lymphosarcoma with more than average reticulin may be virtually indistinguishable by silver impregnation methods. Thus, while an increase of reticulin may help to confirm a diagnosis of reticulum cell sarcoma, a decrease does not exclude it. It must also be remembered that the predominating cell type and the general histological picture may change as the disease progresses. This has been discussed by Custer and Bernhard (1948) and other writers. Such a change may be accompanied by a change in the reticulin content. The most that a study of the gross amount of reticulin can do is to provide confirmatory evidence for a given diagnosis or, occasionally, to suggest an alternative one.

### SUMMARY

1. The reticulum content and pattern in 305 cases of tumours of lymphoid tissue have been examined.

2. The definite but limited value of silver impregnation in the diagnosis of these tumours has been discussed.

I wish to thank Sir Stanford Cade for permission to study many cases under his care. I am indebted to Lt.-Col. P. D. Stewart, R.A.M.C., and W/Cdr. R. M. Cross, R.A.F., for the loan of blocks ; to Springer-Verlag for permission to reproduce Fig. 1 and to the Department of Medical Photography, Westminster Hospital.

### REFERENCES

BAGGENSTROSS, A. H. AND HECK, F. J.—(1940) *Amer. J. med. Sci.*, **200**, 17.
BRILL, N. E., BAEHR, G. AND ROSENTHAL, N.—(1925) *J. Amer. med. Ass.*, **84**, 668.
CALLENDER, G. R.—(1934) *Amer. J. Path.*, **10**, 443.
CUSTER, R. P. AND BERNHARD, W. G.—(1948) *Amer. J. med. Sci.*, **216**, 625.
DUKES, C. E. AND BUSSEY, H. J. R.—(1947) *Brit. J. Cancer*, **1**, 30.
EVANS, WINSTON R.—(1956) ' Histological Appearances of Tumours ', London (Livingstone).
GALL, E. A. AND MALLORY, T. B.—(1942) *Amer. J. Path.*, **18**, 381.
HARRISON, C. V.—(1952) *J. Path. Bact.*, **64**, 513.—(1953), ' Recent Advances in Pathology' 6th Edition, London (Churchill).
JACKSON, H.—(1937) *Surg. Gynec. Obstet.*, **64**, 465.
*Idem* AND PARKER, F.—(1944*a*) *New Engl. J. Med.*, **230**, 1.—(1944*b*) *Ibid.*, **231**, 35.—(1947) ' Hodgkin's Disease and Allied Disorders ', New York (Oxford University Press).
LUMB, G. D.—(1954) ' Tumours of Lymphoid Tissue ', London (Livingstone).
MACKENZIE, D. H.—(1958) *Brit. J. Cancer*, **12**, 14.
MARSHALL, A. H. E.—(1956) ' An Outline of the Cytology and Pathology of the Reticular Tissue ', London (Oliver and Boyd).
PULLINGER, B. D.—(1932) ' Rose Research on Lymphadenoma ', Bristol (John Wright).
RAPPAPORT, R., WINTER, W. J. AND HICKS, E. B.—(1956) *Cancer*, **9**, 792.
ROBB-SMITH, A. H. T.—(1938) *J. Path. Bact.*, **47**, 457.—(1947) ' Recent Advances in Clinical Pathology ', London (Churchill).
SYMMERS, D.—(1938) *Arch. Path.*, **26**, 603.—(1942) *Ibid.*, **34**, 385.
WARREN, S. AND PICENA, J. P.—(1941) *Amer. J. Path.*, **17**, 385.
WETHERBY-MUIR, G., SMITH, P. AND ANDERSON, H. J.—(1952) *Quart. J. Med.*, **21**, 327.